# Bone-targeting carbon dots: effect of nitrogen-doping on binding affinity†

**DOI:** 10.1039/c8ra09729a

**Published:** 2019-01-21

**Authors:** Kyung Kwan Lee, Jae-Geun Lee, Chul Soon Park, Sun Hyeok Lee, Naren Raja, Hui-suk Yun, Jeong-Soo Lee, Chang-Soo Lee

**Affiliations:** Hazards Monitoring BNT Research Center, Korea Research Institute of Bioscience and Biotechnology (KRIBB) Daejeon 34141 Republic of Korea; Disease Target Structure Research Center, Korea Research Institute of Bioscience and Biotechnology (KRIBB) Daejeon 34141 Republic of Korea; Department of Biotechnology, University of Science & Technology (UST) Daejeon 34113 Republic of Korea; Department of Chemical Engineering and Applied Chemistry, Chungnam National University Daejeon 34134 Republic of Korea; Department of Polymer Engineering, Chonnam National University Gwangju 61186 Republic of Korea; Dementia DTC R&D Convergence Program, Korea Institute of Science and Technology (KIST) Seoul 02792 Republic of Korea; Powder and Ceramics Division, Korea Institute of Materials Science (KIMS) Changwon 51508 Republic of Korea

## Abstract

Novel fluorescent carbon dots (CDs) for bone imaging were fabricated *via* a facile hydrothermal method using alendronate in the absence of a nitrogen-doping precursor to enhance bone affinity. One-step synthesized alendronate-based CDs (Alen-CDs) had strong binding activity for calcium-deficient hydroxyapatite (CDHA, the mineral component of bones) scaffold, rat femur, and bone structures of live zebrafish. This was attributed to the bisphosphonate group present on the CD surface, even after carbonization. For comparison, the surface effects of nitrogen-doped CDs obtained using ethylenediamine (EDA), *i.e.*, Alen-EDA-CDs, were also investigated, focusing on the targeting ability of distinct surface functional groups when compared with Alen-CDs. An *in vivo* study to assess the impact on bone affinity revealed that Alen-CDs effectively accumulated in the bone structures of live zebrafish larvae after microinjections, as well as in the bone tissues of femur extracted from rats. Moreover, Alen-CD-treated zebrafish larvae had superior toleration, retaining skeletal fluorescence for 7 days post-injection (dpi). The sustainable capability, surpassing that of Alizarin Red S, suggests that Alen-CDs have the potential for targeted drug delivery to damaged bone tissues and provides motivation for additional *in vivo* investigations. To our knowledge, this is the first *in vitro*, *ex vivo*, and *in vivo* demonstration of direct bone-targeted deliveries, supporting the use of fluorescent CDs in the treatment of various bone diseases such as osteoporosis, Paget's disease, and metastatic bone cancer.

## Abstract 2

Fluorescent carbon dots selectively bind to skull tissues with high affinity, including a strong binding activity for calcium deficient hydroxyapatite, and rat femur, for bone targeted imaging.

## Graphical abstract

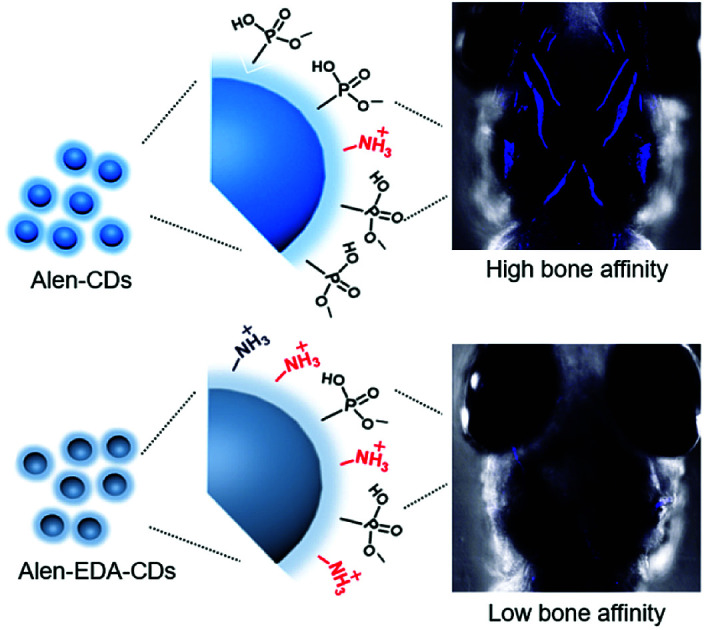

## 1. Introduction

Fluorescent carbon dots (CDs) have recently received considerable attention as important nanomaterials due to their unique properties, namely, higher water solubility, surface modification ability, lower toxicity, and excellent biocompatibility and photostability^1–4^ when compared with traditional semiconductor quantum dots (QDs) and organic dyes. Hence, CDs have a wide variety of promising applications in bioimaging,^5–7^ photocatalysis,^8,9^ optoelectronic devices,^10,11^ drug delivery,^12,13^ and chemical sensor and biosensor technologies.^14,15^ However, the photoluminescence (PL) mechanism of CDs has yet to be established, which has hindered the development and use of CDs as novel materials in applications related to various biological systems.^16^ Several synthesis methods, including top-down cutting and bottom-up dehydration, have been used to fabricate high-quality CDs.^1,17^ Citric acid,^18^ glycerol,^19,20^ amino acid,^21^ ascorbic acid,^22^ and other molecules with abundant hydroxyl, carboxyl, and amine groups have been identified as suitable carbon precursors.^23–27^

Herein, we prepared novel fluorescent CDs *via* a facile hydrothermal method using alendronate as a carbon precursor. Alendronate is widely used in bone disease treatment, bearing a bisphosphonate group with high affinity toward calcium ions related to bone minerals. Among skeletal-related bone diseases, osteoporosis can have fatal consequences and result in major mobility hindrance and mortality.^28^ Currently, there is neither a cure nor the means to efficiently image bone mass. Therefore, treatments have been limited to mitigating bone mass loss, and early detection is critical for positive outcomes. In addition, determining the amount and type of damage in bone tissues is limited to histological studies, but this approach is inherently invasive and only suitable for *in vitro* investigations.^29–33^

Recently, the strong emission of nitrogen-doped fluorescence CDs was confirmed in live zebrafish; these CDs demonstrated long-term stability with no detectable toxicity, suggesting a melanin-binding affinity from strong selective fluorescence at the eyes and melanophore strips in the trunk and tail regions.^34^ Alendronate-conjugated nanoparticles exhibit rapid binding kinetics and strong binding affinity toward hydroxyapatite (HA).^35,36^ In this study, we focused on the bisphosphonate group of alendronate due to its strong affinity toward calcium ions on apatite crystal surfaces;^37^ these surfaces are exposed upon the collapse of the protein–mineral interface when a crack or inelastic deformation is generated.^38^ In addition, bisphosphonates are often used as ligands due to their therapeutic effects in various bone diseases (osteoporosis, Paget's disease, and metastatic bone cancer), as well as their high affinity toward bone tissue.^39,40^ Therefore, it is interesting that the newest trend has focused on CD preparation from “bisphosphonate” raw materials as the carbon source.

The development of novel probes for selective and specific *in vivo* bone imaging is indispensable in the identification, detection, and diagnosis of bone-related developments and dysfunctions. The most widely used bone-imaging techniques are X-ray and computed tomography (CT), but both these techniques expose the human body to potentially harmful ionizing radiation.^41^ In addition, these methods provide only a small snapshot of the sum effect of osteoblast and osteoclast activities in the skeletal structure and do not directly describe cellular events at the time of imaging. While the use of radiolabeled bisphosphonates for bone scintigraphy offers a means to observe site-specific changes in metabolic activity, the use of radioactive agents has limited spatial resolution and does not allow for serial imaging over longer time periods.^42^ Till date, advances in fluorescence instrumentation have provided newer alternatives to traditional bone-imaging methods; however, the development of new *in vivo* bone-imaging fluorescence materials is considerably lagging behind. Moreover, most studies on CDs have primarily focused on their syntheses and applications, while only a few reports have addressed their potential biomedical consequences, let alone the comparative influence of modified/doped CDs.^43,44^

**Scheme 1:**
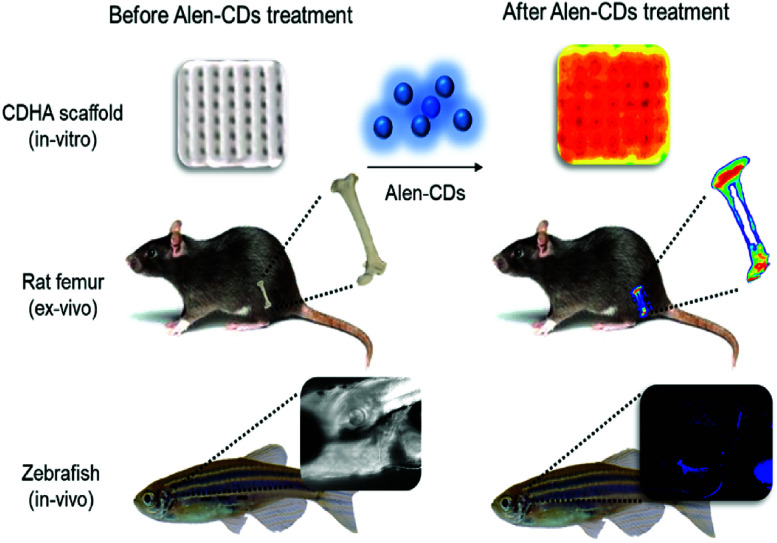
Schematic illustration of fluorescence imaging *via* specific affinity for CDHA scaffolds (*in vitro*), rat femur (*ex vivo*), and live zebrafish (*in vivo*) of Alen-CDs.

In this study, we demonstrated that CDs prepared from alendronate (Alen-CDs) bind to live zebrafish skull tissues with high affinity and selectivity, including a strong binding activity for calcium-deficient hydroxyapatite (CDHA) scaffolds, and rat femur (Scheme 1). Bone-specific binding resulted in strong enhancement of fluorescence that was not observed in other tissues, similar to Alizarin Red S staining as a common fluorescent calcium dye, including noncalcified endochondral elements. The retention of CDs by bones in zebrafish was very stable, long-lasting, and had no detectable toxicity. We investigated the effects of nondoped CDs and nitrogen-doped CDs, which can have different functional groups on their surfaces, depending on the used dopant and surface passivation reagents. Interestingly, a comparison of the bone affinity performances of Alen-CDs generated only from alendronate and CDs generated from alendronate and EDA (Alen-EDA-CDs) as a nitrogen-doping precursor revealed significant differences, likely due to their different crosslinking patterns with EDA. The bone-tissue-targeting results highlighted the potential of Alen-CDs as an emerging fluorescent nanomaterial, without the need for additional surface treatments for specific targeting. These observations support the novel use of CDs as highly specific bioagents for bone imaging, diagnosis, and bone-specific drug delivery vehicles. Further, we hypothesized that such agents will allow microdamage and crack growth to be quantified, and therefore, are useful histological tools in providing data for modeling the material behavior of the bone.

## 2. Experimental

### 2.1. Materials

Alendronate sodium trihydrate was purchased from Tokyo Chemical Industry Co. Ltd. (TCI). Ethylenediamine (EDA; 99.5% purity), calcium carbonate, calcium phosphate, pyrophosphate, calcium oxide, Alizarin Red S, and deuterium oxide were purchased from Sigma Aldrich (St. Louis, MO, USA). All the other reagents were of the analytical grade and used without further purification. Double deionized water used throughout the study was prepared using a Milli-Q system (Millipore, Bedford, MA, USA). The cell fixation and permeabilization solution were purchased from BD (Cytofix/Cytoperm). CDHA scaffolds and normal rat femurs (8 weeks, male, Sprague-Dawley) were supplied by the Korea Institute of Materials Science (KIMS) and Bio-Evaluation Center at KRIBB, respectively.

### 2.2. Instruments

UV-vis absorption and fluorescence spectra were obtained using a Beckman-Coulter DU800 spectrophotometer (Pasadena, CA, USA) and Scinco FluoroMate FS-2 spectrometer (Seoul, South Korea), respectively. The morphology and microstructure of the CDs were examined using high-resolution transmission electron microscopy (HR-TEM) on a Tecnai G2 F30 S-TWIN microscope (FEI Company) with an accelerating voltage of 300 kV. The FT-IR spectra were recorded with an Alpha-P Bruker optics FT-IR spectrometer (Bruker Corp., Billerica, MA, USA). X-ray photoelectron spectrometer (XPS) spectra were analyzed by a Thermo Fisher Scientific MultiLab 2000 (Thermo Fisher, Waltham, MA, USA) with monochromatic X-ray source Al-Kα excitation (1486.6 eV). Energy-dispersive X-ray (EDAX) spectra were obtained using a JEOL 6700F SEM (JEOL, Ltd., Tokyo, Japan) using a 20 kV electron beam and an EDAX Genesis 4000 X-ray analysis system as a detector. The fluorescence lifetime was measured with a Horiba FM-4P time-correlated single-photon counting system (Horiba Ltd., Kyoto, Japan). The fluorescence decay curves were recorded using 375 nm excitation and an emission wavelength of 518 nm. Phosphorus nuclear magnetic resonance (^31^P-NMR) data were measured by nuclear magnetic resonance spectroscopy at 600 MHz (Bruker BioSpin, Avance III). Cell permeability analysis and live zebrafish imaging were captured by using confocal laser scanning microscopy (Olympus FV 1000, Olympus Corp., Tokyo, Japan). Affinity tests for rat femur specimens were conducted using an LAS-4000 luminescent image analyzer (Fujifilm Holdings Corp., Tokyo, Japan).

### 2.3. Synthesis of CDs

Alen-CDs and Alen-EDA-CDs were hydrothermally synthesized using alendronate as the carbon source, with and without EDA. Briefly, 1 g of alendronate sodium trihydrate was dissolved in 50 mL of deionized water and stirred at 300 rpm for 2 h. The solution was then transferred to a Teflon autoclave. After heating at 180 °C for 12 h, the autoclave was allowed to naturally cool to room temperature. The obtained mixtures were purified using a 0.2 μm syringe filter and dialysis against deionized water (MWCO: 100–500 Da) to remove excess alendronate, EDA, and byproducts at 120 rpm for 3 days. The clear solution was lyophilized. The yellow powder was collected for characterization. To generate nitrogen-doped or amine-passivated CDs, 50 mL of alendronate stock solution was poured into a beaker and then 1 mL of EDA was added. The procedure used to obtain Alen-EDA-CDs was similar to that of Alen-CDs. The final Alen-EDA-CD product was a slightly viscous yellow powder.

### 2.4. Determination of fluorescence quantum yields (QYs)

The fluorescence QYs for Alen-CDs and Alen-EDA-CDs were determined using anthracene (*Φ*_F_ = 0.27 in ethanol) as the fluorescent standard. The QYs were calculated using the following equation:*Φ*_F_(X) = *Φ*_F_(S)(*A*_S_*F*_X_/*A*_X_*F*_S_)(*n*_X_/*n*_S_)^2^where *Φ*_F_ is the fluorescence QYs, *A* is the absorbance at the excitation wavelength, *F* is the area under the corrected emission curve, and *n* is the refractive index of the solvents used. The subscripts S and X refer to the standard and unknown, respectively.

### 2.5. Cellular imaging and cytotoxicity

HeLa cells were cultured in Dulbecco's Modified Eagle's Medium (DMEM, Gibco) supplemented with 10% fetal bovine serum (FBS, Gibco) and 1% penicillin/streptomycin (Gibco) in a humidified incubator with 5% CO_2_ in air at 37 °C. The seeding density of HeLa cells was 2.0 × 10^6^ cells per dish in 10 mL DMEM. The cells were seeded in an 8-well plate to obtain live cell images at 1.0 × 10^4^ cells per well density. After 24 h, the cells were rinsed slightly with Dulbecco's phosphate-buffered saline (DPBS) to remove the media. Then, the cells were incubated with Alen-CDs and Alen-EDA-CDs (100 μg mL^−1^) in the medium for 4 h. Prior to imaging, the cells were washed three times with DPBS and attached using fixation and permeabilization solutions. The fluorescence images were acquired using confocal microscopy (Olympus FLUOVIEW FV1000). Then, the cell subculture was used to test the cytotoxicity in flat-bottomed 96-well plates at a density of 0.05 × 10^6^ cells per well in 200 μL of complete media (DMEM). After the cells were incubated for 1 day, Alen-CDs and Alen-EDA-CDs were treated to the cellular sample (96-well plate) for 1 h at different concentrations (100–500 μg mL^−1^). After incubation for 24 h, 10 μL of CCK-8 solution (Dojindo, Japan) was added to each plate well, and the cells were further incubated for 30 min. The absorbance at 450 nm was measured with a microplate reader (SpectraMax M2, Molecular Devices, San Jose, CA, USA).

### 2.6. *In vitro* bone affinity test

The *in vitro* binding capabilities of Alen-CDs and Alen-EDA-CDs on CDHA scaffolds and rat femurs were evaluated using a fluorescence imaging system (Fujifilm LAS-4000, Japan). Flatly sliced rat femurs were obtained from a medical device manufacturing company (Genoss Co., Ltd) to carry out fluorescence measurements under the same conditions. The as-prepared CDHA scaffolds and sliced rat femurs were incubated in 300 μg mL^−1^ Alen-CDs and Alen-EDA-CDs aqueous solutions, respectively, mildly shaken at 37 °C overnight. After washing three times using deionized water, tests to determine the affinity of Alen-CDs and Alen-EDA-CDs toward CDHA scaffolds and sliced rat femurs were carried out.

### 2.7. Live confocal imaging of zebrafish larvae

Wild-type adult zebrafish (AB line) were maintained by following the standard procedures (http://www.zfin.org) of a 14 h light/10 h dark cycle at 28 °C. The embryos were obtained by natural spawning of the adult AB line. AB-line embryos were maintained in E3 egg water (5 mM NaCl, 0.17 mM KCl, 0.33 mM CaCl_2_, and 0.33 mM MgSO_4_) in a Petri dish at 28.5 °C. Zebrafish husbandry and animal care were performed in accordance with the guidelines from the Korea Research Institute of Bioscience and Biotechnology, as approved by KRIBB-IACUC (approval number: KRIBB-AEC-18128). Prior to nanoparticle and Alizarin Red S microinjections, the fish were anesthetized using a tricaine solution and lined up on a 1.5% agarose gel plate in a Petri dish. Approximately 5 nL of Alen-CDs and Alen-EDA-CDs (15 μg μL^−1^) were injected into the abdominal cavity of the zebrafish larvae on 6 days post-fertilization (dpf). To identify the mineralized bone, approximately 5 nL of Alizarin Red S (300 ng μL^−1^) was injected into the same place as the positive control. Injected zebrafish larvae were transferred to the plates with flash E3 egg water and reared at 28.5 °C. At 2 h and 7 days post-injection, live specimens were embedded onto glass-bottomed imaging dishes with 1.5% low-melting-point agarose. Agarose was overlaid with E3 egg water with tricaine. The head skeleton of the zebrafish larvae was imaged using an Olympus FV1000 confocal microscope. Three independent experiments with 20 zebrafish larvae per condition were carried out.

## 3. Results and discussion

### 3.1. Characterization of Alen-CDs and Alen-EDA-CDs

**Scheme 2:**
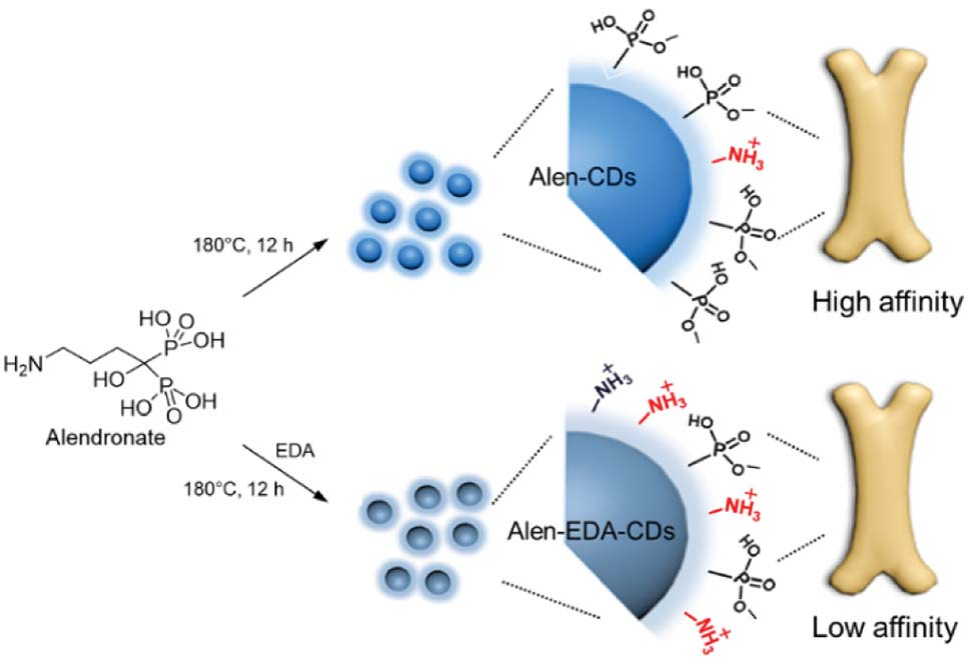
Schematic illustration of the synthesis and bone affinity of Alen-CDs and Alen-EDA-CDs.

**Fig. 1:**
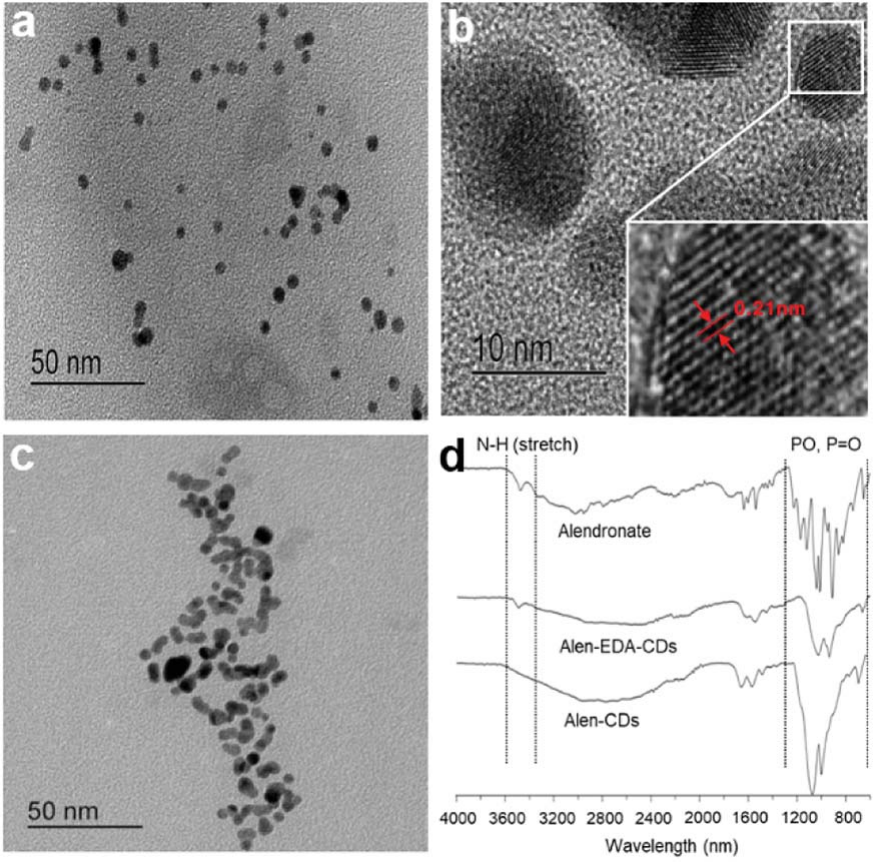
TEM images of (a and b) Alen-CDs, (c) Alen-EDA-CDs, and (d) FT-IR spectra of alendronate, Alen-CDs, and Alen-EDA-CDs.

Scheme 2 shows a schematic illustration of Alen-CDs and Alen-EDA-CDs syntheses. To evaluate the optical and surface properties depending on the nitrogen doping agent, Alen-CDs and Alen-EDA-CDs were synthesized with and without the use of EDA. Among the Alen-EDA-CDs prepared from the addition of EDA at various weight ratios (335–2000 μL), “sample d” had the highest fluorescence intensity in water and was selected for further characterization (Table S1†). While it is currently not possible to describe the specific atomic structure of CDs, various characterization methods were applied in this study to reveal different aspects about the CD structure. Transmission electron microscopy (TEM) was used to determine the morphologies of both Alen-CDs and Alen-EDA-CDs, as shown in Fig. 1. Alen-CDs and Alen-EDA-CDs exhibited crystalline and amorphous nanostructures that were made up of sp^2^ and sp^3^ carbon atoms at the edges of the defect sites as amorphous structures. Alen-CDs had individual nanoparticles (diameter: ∼5 nm) with well-graphitized stacking layers. High-resolution TEM images revealed well-resolved lattice fringes with interplanar spacings of 0.21 nm, close to the (020) and (100) diffraction facets of graphite carbon, confirming the crystalline structure (Fig. 1b).

In contrast, the crystallinity of Alen-EDA-CDs was relatively lower than that of Alen-CDs, with some aggregation and no uniformity in size (Fig. 1c). These observations indicate that the variation in the surface functional groups according to EDA use induced a remarkable influence on the morphology.^45^ Fourier-transform infrared (FT-IR) spectroscopy, XPS, and ^31^P NMR measurements were conducted to identify functional group variations on the CD surfaces. The FT-IR spectrum of alendronate revealed the characteristic absorptions of amine at 3476.21 cm^−1^ and the phosphorous stretching region at 1043.6, 1014.63 (*V*_s_, *V*_as_ of PO_3_), and 912.83 cm^−1^ (*V*, POH), as shown in Fig. 1d.^46^ In contrast, the characteristic peaks of Alen-EDA-CDs and Alen-CDs, as compared to alendronate, revealed broad absorption bands around 1200–900 cm^−1^, indicating that bisphosphonate groups were associated with other heteroatoms and transferred from alendronate molecules to the CD surface during CD formation. Although the FT-IR spectra of Alen-CDs and Alen-EDA-CDs exhibited similar features, differences were observed in the spectrum attributable to the amine group. The IR absorption peak at 3500 cm^−1^ of Alen-EDA-CDs originates from the stretching vibrations of N–H groups, indicating the stretching of the crosslinked amine group *via* reaction with EDA and the relatively abundant amine group on Alen-EDA-CDs when compared with Alen-CDs. These characteristic peaks confirm the successful fabrication of EDA-dependent CDs, with a negligible amine group for Alen-CDs.

**Fig. 2:**
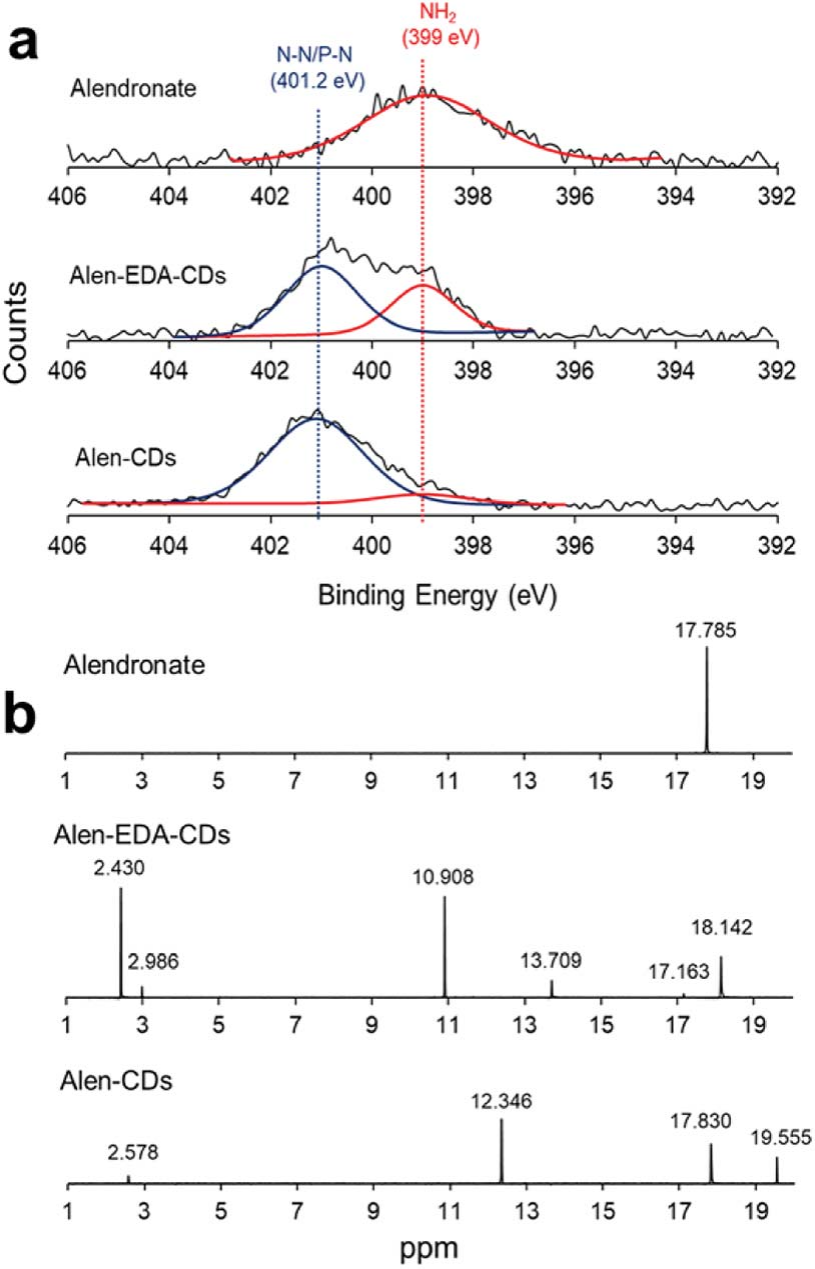
(a) XPS spectra for N_1s_ and (b) ^31^P-NMR spectra.

Next, we attempted to determine the surface functional state and components of Alen-CDs and Alen-EDA-CDs using XPS spectra. As shown in Fig. 2a, alendronate has a single peak for NH_2_ at 399 eV. However, new emerging peaks in the N_1s_ were observed at 401.2 eV, indicating the presence of N–P/N–N bonds in Alen-CDs and Alen-EDA-CDs. The NH_2_ peak of Alen-EDA-CDs was remarkably higher than that of Alen-CDs due to the EDA doping effect. The full narrow spectra of C_1s_, N_1s_, and P_2p_ are shown in Fig. S1.† For alendronate only, C_1s_ and N_1s_ features were observed at the peak positions of 286, 285, and 399 eV, indicative of C–O, C–C/C–N/C–P, and NH_2_ groups, respectively (Fig. S1a†); however, the P_2p_, C_1s_, and N_1s_ spectrum of Alen-EDA-CDs were assigned to the 133.1, 134, 286, 285, 284.2, 401, and 399 eV, which indicated P–O/P=O, P–N, C–O, C–C/C–N/C–P, C=C, N–N/N–P, and NH_2_ groups, respectively. No C=C peak was observed in alendronate only (Fig. S1a†). Similarly, the P_2p_, C_1s_, and N_1s_ peaks of Alen-CDs were observed at 133.1, 132.9, 286, 285, 284.5, 401, and 399 eV, which were assigned to P–O/P=O, P–N, C–O, C–C/C–N/C–P, C=C, N–N/N–P, and NH_2_, respectively,^47^ revealing a higher intensity for the NH_2_ peak of Alen-EDA-CDs by EDA doping when compared with that of Alen-CDs (Fig. S1c†).

Consequently, the XPS results confirmed that the bisphosphonate groups in alendronate covalently bonded to the heteroatoms during CD formation. However, the P_2p_ peak of alendronate was observed in a single peak assigned to the P–O/P=O groups at 133.3 eV; the P_2p_ peaks of Alen-EDA-CDs and Alen-CDs were assigned to P–C and P–N bonding at 134 and 132.9 eV, respectively. These newly emerged peaks are associated with the intermolecular bonding between the amine and bisphosphonate groups of alendronate. ^31^P-NMR spectroscopy provides a qualitative analysis of the phosphorus hybridization state inside both Alen-CDs and Alen-EDA-CDs, which can be the key tool in differentiating mono-, di-, and tri-phosphoesters *via* characteristic chemical shifts. As shown in Fig. 2b, the phosphorous atom of the bisphosphonate group in the alendronate molecule can be assigned to a single peak at ∼17.785 ppm. In contrast, both Alen-CDs and Alen-EDA-CDs exhibited not only various chemical shifts in dispersion and differentiation but also an obvious difference in phosphorous atoms. When compared with Alen-EDA-CDs, the ^31^P-NMR spectrum of Alen-CDs show the existence of a peak at ∼17.830 ppm, which corresponds to the phosphorous peak of alendronate, with no noticeable peak shift. The existence of a bisphosphonate group in Alen-CDs indicates that the bisphosphonate group of the alendronate precursor molecule remains partially intact even after CD formation, increasing the possibility of efficient targeting ability for bone tissues. The newly emerged peak at ∼10.908 ppm for Alen-EDA-CDs can be attributed to the phosphorous atom in the phosphoramidate group (–PO_3_–NH_2_) formed by alendronate and EDA crosslinking. For EDA doping during CD formation, the resultant Alen-EDA-CDs showed several peaks, as well as a significant reduction in the phosphorous peak intensity of the bisphosphonate group (∼17.163 ppm). Moreover, the ^31^P-NMR analysis revealed that Alen-CDs retained relatively abundant bisphosphonate groups on their surfaces. Surface charge differences between Alen-CDs and Alen-EDA-CDs were determined using zeta potential analyses. As shown in Fig. S2,† Alen-CDs had a negative charge value of −12.68 mV due to the abundant bisphosphonate groups, whereas Alen-EDA-CDs had a positive charge value of +6.93 mV, caused by the relatively rich amine groups on the surface. A difference in the amine and bisphosphonate group distributions on the surface determines the affinity of CDs toward bone tissue, as shown in Scheme 2.

Therefore, the higher affinity of Alen-CDs resulted in high fluorescence intensities for CDHA scaffolds, as well as the bone of live zebrafish larvae.

### 3.2. Optical properties of Alen-CDs and Alen-EDA-CDs

**Fig. 3:**
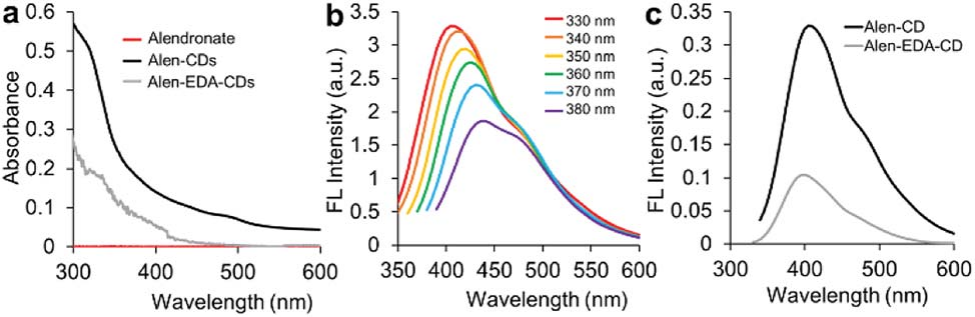
(a) UV-vis spectra of alendronate, Alen-CDs, and Alen-EDA-CDs; (b) fluorescence spectrum of Alen-CDs; and (c) fluorescence intensities of Alen-CDs and Alen-EDA-CDs.

EDA, with its short chains, is often used as a passivating agent. Therefore, amine groups may be effective in controlling the surface passivation of CDs to determine the PL properties. Fig. 3a shows the absorption spectra of Alen-CDs and Alen-EDA-CDs, exhibiting broad absorption bands. Because alendronate exhibits no emission in the visible (vis) and near-ultraviolet (UV) range, the bright PL emission should, therefore, be attributed to the formed CDs. A broad UV-vis absorption shoulder is located between 300 and 350 nm, which is typically assigned to the π–π* transition of the aromatic sp^2^ domains from the carbon core.^48^ When the Alen-CDs were excited over the wavelength range of 330 to 380 nm, the corresponding emission peaks concomitantly shifted from 400 to 460 nm, while becoming weaker and gradually red-shifted (Fig. 3b); this type of excitation-dependent emission has been demonstrated in other CD studies.^17^ These PL properties of CDs are determined by the radiative recombination of excitons,^49^ particle size distribution, surface traps, and quantum effects.^23^ However, till date, the exact mechanism has yet to be established, and the requirements for surface passivation to induce fluorescence emission are poorly understood.^48^ Fig. S3† shows the PL behavior as a function of the surface state for various EDA concentrations (0.335, 0.5, 1, and 2 mL). As the EDA concentration increased, the red-shift increased marginally, implying a variation in the passivation degree of surface traps on CDs by changing their surface coverage with amine groups, *i.e.*, an increase in nitrogen content. The CDs prepared at lower EDA concentrations had fewer surface traps; therefore, their luminescence was excitation-dependent. In contrast, at higher concentrations, CDs were fully surface-passivated and independently emitted depending on the excitation wavelength. As expected, Alen-CDs prepared without EDA exhibited lower excitation dependence than those of CDs prepared with EDA.^50,51^ Regardless of the use of EDA, alendronate not only acted as the carbon source but also provided amine- and bisphosphonate groups on the CD surface, thereby passivating the surface traps. The excellent excitation-dependent luminescence of the as-prepared Alen-CDs, which were rich in bisphosphonate groups, was tested for various phosphate-related functions. Hence, we deduced that the amine groups induce excitation-dependent luminescence.

The QYs of Alen-CDs and Alen-EDA-CDs were measured to be 13.5% and 10.6%, respectively, when compared with that of anthracene as the reference fluorescence dye (Fig. S4†). Generally, CDs possess tunable emissions even without any surface passivation; however, they usually have very low QYs due to unstable surface defects, leading to reduced radiative recombinations. Nonetheless, the higher QYs of Alen-CDs than Alen-EDA-CDs indicate surface defect stabilization and strong fluorescence emission. The QYs increase with a decrease in the amount of amine groups, with Alen-CDs having the highest degree of surface oxidation.^52^ Similarly, this observation was also confirmed in the twofold higher fluorescence intensity of Alen-CDs when compared with that of Alen-EDA-CDs (Fig. 3c). Based on these findings, we speculate that the excitation dependence of CD luminescence can be controlled by engineering the abovementioned surface states of CDs, as shown in Fig. S3.† The fluorescence lifetimes for both types of CDs were measured in triplicate using a 375 nm laser as the excitation source; lifetimes of 4.43 ns (Alen-CDs) and 3.64 ns (Alen-EDA-CDs) were obtained (Fig. S5†). Both CD samples displayed multiexponential fluorescence decay and excellent stability. In contrast to typical organic fluorophores (*e.g.*, Cy5 (*τ*_av_ = 1.5), Nile Red (*τ*_av_ = 3.6), and fluorescein (*τ*_av_ = 4.0), including Alen-EDA-CDs), Alen-CDs exhibited a longer experimental fluorescence decay time in water. This allows the temporal discrimination of signals from cellular auto-fluorescence and scattered excitation light by time-gated measurements, thereby enhancing measurement sensitivity.^53^

### 3.3. Cytotoxicity and live cell imaging

**Fig. 4:**
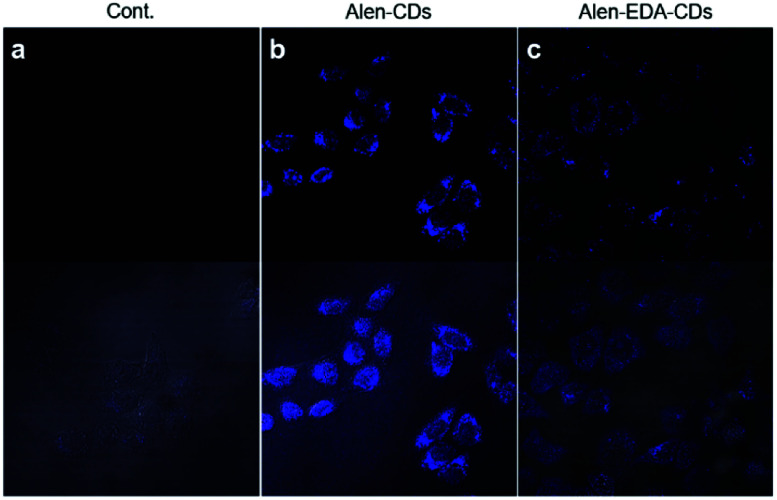
Confocal microscopy images (60×, ex; 405 nm/em; 440–470 nm) of HeLa cells treated with 300 μg mL^−1^ Alen-CDs and Alen-EDA-CDs: (a) control group, (b) Alen-CDs, and (c) Alen-EDA-CDs.

To establish the potential efficacies of Alen-CDs and Alen-EDA-CDs for biomedical imaging applications, we evaluated the permeability and cytotoxicity in live cells using a fluorescence cell imaging system. The cell permeabilities of Alen-CDs and Alen-EDA-CDs were investigated by treating HeLa cells with 300 μg mL^−1^ of each CD type. Confocal microscopy under 405 nm laser excitation was used to evaluate the cell permeability levels. HeLa cells exhibited a blue color upon excitation, whereas no visible fluorescence was detected in the control cells (Fig. 4a) without incubation with CDs under the same conditions. The emission was largely located in the cytoplasmic regions, particularly around the cell nucleus. This finding suggests that Alen-CDs can pass though HeLa cell membranes and enter into cells. Confocal laser scanning confocal microscopy revealed that Alen-CDs were ideal for bioimaging due to the remarkably high photostability in living cells and good biocompatibility, with no blinking and low photobleaching.

In contrast, Alen-EDA-CDs had relatively low fluorescence intensities. The permeabilities of Alen-CDs and Alen-EDA-CDs were comparable; however, we observed a difference between the two CD types in their cellular cytotoxicity levels. Fig. S6† shows that Alen-CDs had remarkably low cytotoxicity, with cells retaining a viability of >80–90% over a concentration range of 100–500 μg mL^−1^ for 24 h. In contrast, Alen-EDA-CDs had <80% cell viability with concentrations within 200–500 μg mL^−1^. These results suggest that the relatively rich amine group exposed on the Alen-EDA-CDs surface may increase the toxicity of CDs,^54^ which is in good agreement with our expectations of increased nitrogen-doping of the CD surface, as discussed earlier.

### 3.4. *In vitro* binding affinity toward CDHA scaffolds

After confirming good stability and biocompatibility and a high specificity of Alen-CDs, the binding activity and fluorescence response of Alen-CDs were quantitatively evaluated by estimating the affinity for CDHA scaffolds as the model bone. Because HA—constructed from type-I collagen—is the main component of bone tissues, the binding activity of HA for Alen-CDs was assessed for bone targeting. Several studies have reported that the macromolecular conjugates based on alendronate or acidic peptide specifically bind to HA; the binding amount does not change significantly over time either *in vitro* or *in vivo*.^55,56^ This strong affinity of alendronate is the key to our approach. The specificity of Alen-CDs for HA was verified over other calcium salts with fluorescence imaging by mixing Alen-CDs and calcium salts and soaking them in PBS. Most of the Alen-CDs were bound to the CDHA powder (77.50%), whereas only negligible amounts of calcium carbonate (CC) (3.51%), calcium phosphate (CP) (3.59%), calcium pyrophosphate (CPP) (3.53%), and calcium oxalate (CO) (3.20%) were nonspecifically adsorbed (Fig. S7†). The improved binding affinity for CDHA can be attributed to salt formation by the reaction of Ca^2+^ on CDHA with the bisphosphonate group on the surfaces of Alen-CDs.^57,58^ As such, we expected that Alen-CDs could be immobilized on bone tissues and retain their bioimaging properties *in vivo*.

**Fig. 5:**
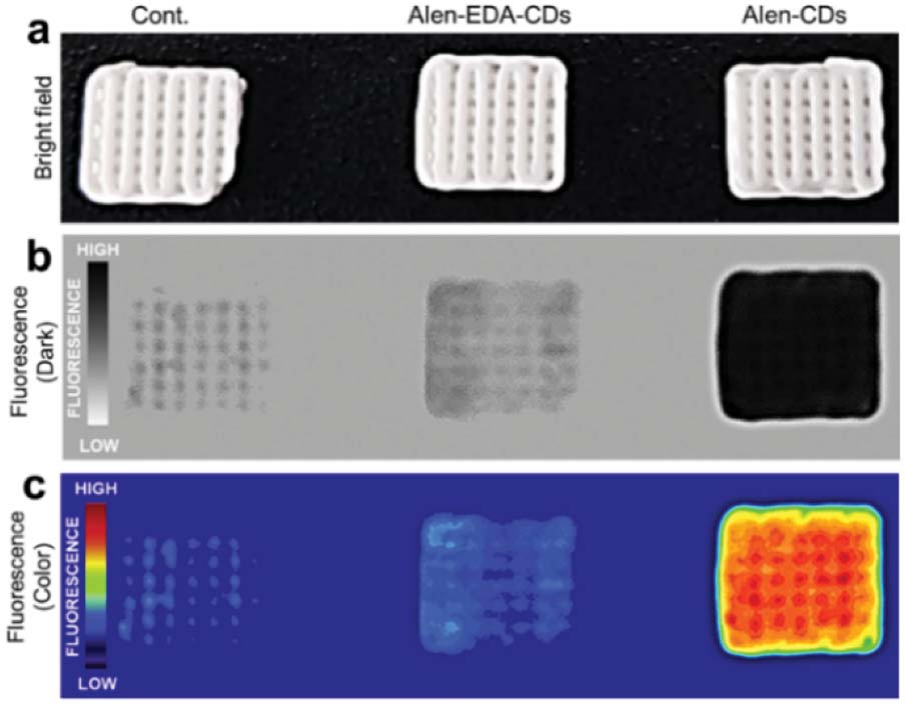
Fluorescence images of CDHA scaffolds treated with Alen-CDs (300 μg mL^−1^) and Alen-EDA-CDs (300 μg mL^−1^) at 37 °C for 12 h.

To explore this possibility, Alen-CDs were administered to CDHA scaffolds, which were fabricated as the model bone using a three-dimensional (3D) printer. The CDHA scaffold fabrication strategy is shown in Fig. S8.† The α-tricalcium phosphate (α-TCP) paste exhibited sufficient fluidity, stability, and workability for printing applications, involving the stacking of 3D structures using pulsed-electron-beam deposition (PED). An α-TCP scaffold green body, with computer-controlled architecture and pore conditions, was successfully fabricated and then immersed in the PBS solution to solidify the green scaffolds with the cement reaction. X-ray diffraction (XRD) results of the scaffold green body were equivalent to those of the α-TCP powder. The XRD pattern revealed a dramatic change in the CP crystal structure after cementing α-TCP to CDHA (Fig. S9a†). The Alen-CD-incubated CDHA scaffolds were fabricated by exploiting the strong affinity between the Alen-CD bisphosphonate group and the scaffold surface. The adsorption of Alen-CDs on the scaffold was confirmed from a significant decrease in the fluorescence intensity of the remaining Alen-CDs solution after incubation (Fig. S10a†) and the fluorescent intensity of CDHA scaffolds (Fig. S10b†). In the EDAX analysis, carbon and nitrogen were only observed after Alen-CDs treatment, implying the specific binding of Alen-CDs on the CDHA scaffold surface (Table S2†). CDHA scaffolds incubated for 12 h in Alen-CDs revealed dose-dependent increases in fluorescence at 0, 100, and 300 μg mL^−1^ (Fig. S10b†). A difference in the concentration of Alen-CDs attached to the CDHA scaffolds was detected *via* changes in the fluorescence intensities. As shown in Fig. 5, the specific binding ability of Alen-CDs to CDHA scaffolds was demonstrated by the relatively low fluorescence signal of the Alen-EDA-CDs-labeled scaffold. As expected, this significant difference resulted from the distinct surface properties of both Alen-CDs and Alen-EDA-CDs, influencing the binding affinity when the functional groups of the CDs approached the scaffold surface.

### 3.5. *Ex vivo* and *in vivo* bone imaging

**Fig. 6:**
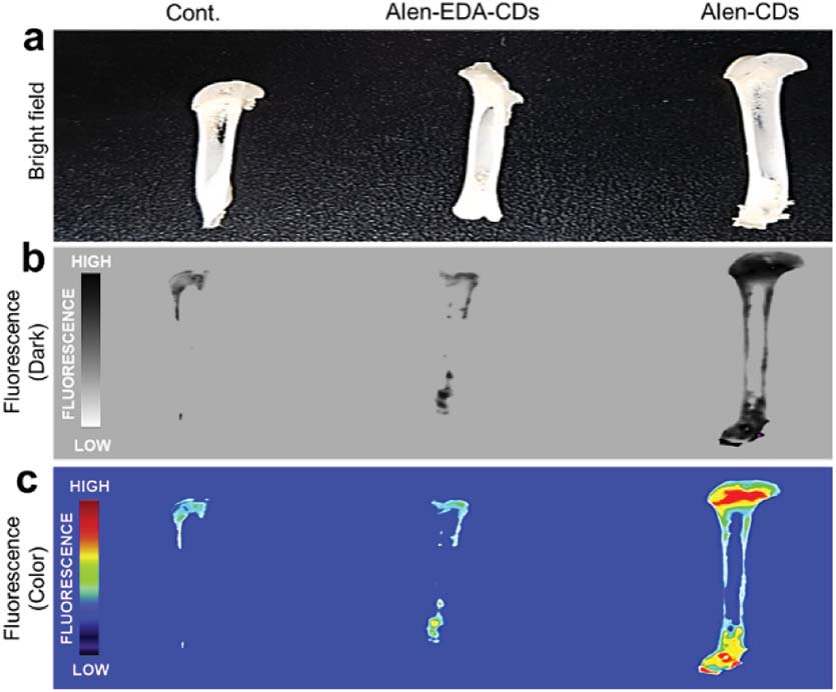
Fluorescence images of rat femurs treated with Alen-CDs (300 μg mL^−1^) and Alen-EDA-CDs (300 μg mL^−1^) at 37 °C for 12 h.

To evaluate the targeting capabilities for the bone tissue of Alen-CDs and Alen-EDA-CDs, rat femurs and live zebrafish larvae were used for *in vitro* and *in vivo* imaging, respectively. First, rat femur bone specimens were excised, sectioned, sliced, and finally soaked in Alen-CDs and Alen-EDA-CDs solutions for imaging *via* fluorescent microscopy. Alen-CD-treated femurs exhibited a very high level of fluorescence intensity when compared with those of the control and Alen-EDA-CDs groups (Fig. 6). The discrepancies in the *in vitro* and *ex vivo* results may be attributed to the large differences in the surface properties of (bisphosphonate-group-rich) Alen-CDs and (bisphosphonate-group-poor) Alen-EDA-CDs.

Further, these observations suggest that the targeted labeling of bone tissues by bisphosphonate-functionalized Alen-CDs was due to the calcium specificity of the molecular groups and not due to an electrostatic association or increased surface roughness of the damaged tissue. Therefore, both the visual observations provided qualitative evidence for the targeted labeling of bone tissue by functionalized Alen-CDs and suggest that bisphosphonate-functionalized Alen-CDs provide superior binding affinity, which is consistent with the previous experiments involving binding toward CDHA scaffolds.

**Fig. 7:**
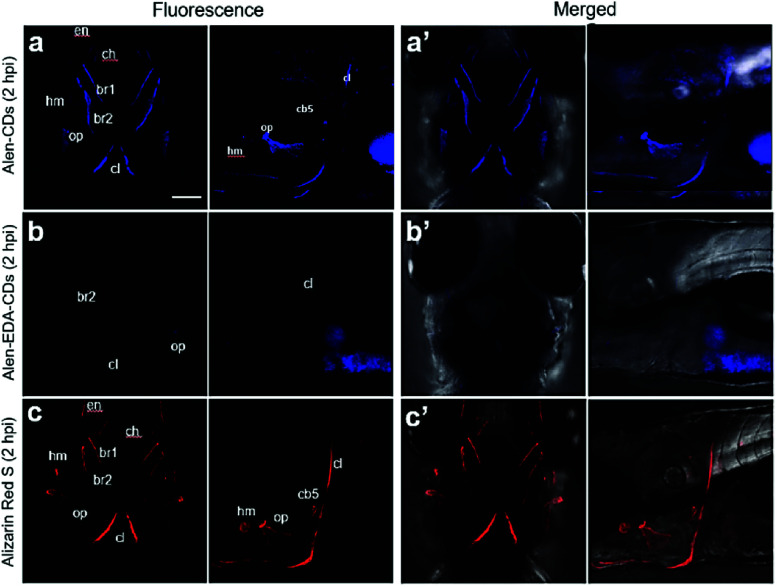
Confocal microscopy images for 6 dpf head skeleton of zebrafish larvae treated with (a and a′) Alen-CDs, (b and b′) Alen-EDA-CDs, and (c and c′) Alizarin Red S. Abbreviations of bones: hyomandibula (hm), opercle (op), ceratobranchial 5 (cb5), cleithrum (cl), entopterygoid (en), branchiostegal ray1 (br1), branchiostegal ray2 (br2). Scale bar = 100 μm.

Zebrafish larvae have been of particular interest in this study and modeling of human diseases due to their rapid development, optical clarity, and shared anatomical and physiological characteristics.^59–63^ Alizarin Red S has long been used to stain the mineralized skeleton in fixed specimens from all the vertebrate groups. The correct evaluation of mineralization is fundamental to the study of skeletal development, maintenance, and regeneration. Current methods to visualize mineralized tissues in zebrafish rely on (1) fixed specimens, (2) radiographic and μCT techniques that are ultimately limited in resolution, and (3) vital stains with fluorochromes that are indistinguishable from the signal of green fluorescent protein (GFP)-labeled cells. To investigate the specific affinity of Alen-CDs for bones, we abdominally injected Alen-CDs, Alen-EDA-CDs, and Alizarin Red S, which is used as a positive control bone-specific imaging reagent, into live zebrafish larvae. The fluorescence images of the head skeleton of live zebrafish larvae were observed at 2 h post-abdominal injection (2 hpi) of the sample solutions (15 μg μL^−1^) at 6 dpf. As shown in Fig. 7a and a′, Alen-CDs exhibited strong fluorescence, selectively in the head skeleton of zebrafish larvae, indicating a bone-tissue-dependent high affinity. In contrast, the fluorescence emission in Alen-EDA-CDs at 6 dpf was very weak, exhibiting relatively low affinity and specificity (Fig. 7b and b′). As a positive control agent that identifies mineralized bones, clear labeling of the Alizarin Red S was also observed in the head skeleton with high specificity, similar to that observed in Alen-CD-treated zebrafish larvae (Fig. 7c and c′). Moreover, all the parts stained by Alen-CDs perfectly corresponded with the stained parts using Alizarin Red S. This finding suggests a strong interaction between the functional groups on the Alen-CD surface and bone tissue. Further, the Alen-CD-treated zebrafish larvae exhibited superior toleration toward binding affinity, retaining skeletal fluorescence at 7 days post-abdominal injection (dpi)—the experiment's end point (*n* = 30; 95% survival; 5 nL injection from a 5 mg mL^−1^ Alen-CDs suspension).

**Fig. 8:**
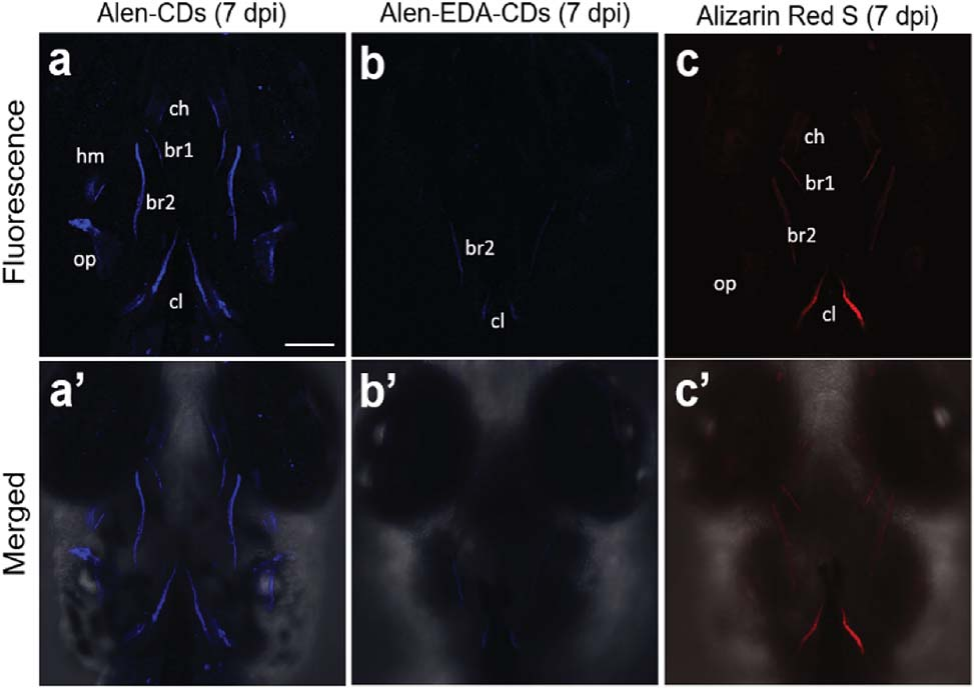
Confocal microscopy images of zebrafish larvae with (a and a′) Alen-CDs, (b and b′) Alen-EDA-CDs, and (c and c′) Alizarin Red S at 7 dpi. Abbreviations of bones: hyomandibula (hm), opercle (op), cleithrum (cl), entopterygoid (en), branchiostegal ray1 (br1), branchiostegal ray2 (br2).

The sustainable capability of the bones stained with Alen-CDs was evaluated 7 dpi for Alen-CDs, Alen-EDA-CDs, and Alizarin Red S. Fig. 8 shows the confocal microscopy images of zebrafish larvae at 7 dpi with Alen-CDs (15 μg μL^−1^) at 6 dpf to confirm sustainability and biocompatibility. The images revealed that the injected Alen-CDs (Fig. 8a and a′) had high affinity and specificity in the jawbones of zebrafish larvae, but Alen-EDA-CDs did not (Fig. 8b and b′). The fluorescence intensity and fluorescent sites in the skeletal structures and cleithrum were the same as those immediately after injection in only Alen-CD-stained bone tissues. Therefore, the fluorescence of Alen-CDs was strongly preserved post-injection; however, the fluorescence intensity of Alizarin Red S-stained bone tissues decreased distinctly at the jawbone when compared with that of 2 hpi (Fig. 8c and c′). These results also confirmed that Alen-CDs bind to calcified bones, surpassing the sustainable capability of Alizarin Red S, due to the functional group with higher affinity toward bone tissues.^37^ In addition, surface modification *via* the nitrogen-doping of Alen-EDA-CDs was ineffective with regard to the binding affinity toward bone tissue. Together, these results show that Alen-CDs are nontoxic, while having high affinity and specificity for calcified bones, clearly confirming the superior targeting efficiency with regard to bone tissues and their use in bone-targeted delivery systems. Therefore, the investigation of various bone-related diseases may benefit from the development of novel bone-targeting Alen-CDs.

## 4. Conclusions

In summary, we successfully fabricated bone-targeting CDs based on alendronate (Alen-CDs) and demonstrated their photostability, cell permeability, and low cytotoxicity for bone imaging. The *in vivo* study of bone affinity revealed that Alen-CDs effectively accumulated in the bone structures of live zebrafish larvae after microinjections, as well as in the bone tissues of femur extracted from rats, with high affinity and selectivity. These results were attributed to the bisphosphonate group present on the surfaces of Alen-CDs, even after carbonization. The sustainable capability, surpassing that of Alizarin Red S, suggests that Alen-CDs have the potential for targeted drug delivery into damaged bone tissues and provides motivation for additional *in vivo* investigations. In conclusion, this is the first *in vitro*, *ex vivo*, and *in vivo* demonstrations of direct bone-targeted deliveries supporting the use of fluorescent CDs in the treatment of various bone diseases such as osteoporosis, Paget's disease, and metastatic bone cancer.

## Conflicts of interest

The authors declare no competing financial interest.

## Supplementary Material

RA-009-C8RA09729A-s001
