# Supplementary material for: Bone-targeting carbon dots: effect of nitrogen-doping on binding affinity
Source: RSC Adv. 2019 Jan 21;9(5):2708–17. doi: 10.1039/c8ra09729a (PMC9059868; doi:10.1039/c8ra09729a)
Supplement: RA-009-C8RA09729A-s001 [file RA-009-C8RA09729A-s001.pdf]

Electronic Supplementary Material (ESI) for RSC Advances.
This journal is © The Royal Society of Chemistry 2019

Supporting Information

Bone-targeting carbon dots : effect of binding affinity by nitrogen-doping

Kyung Kwan Lee,§[a,d] Jae-Geun Lee,§[b,c] Chul Soon Park,[e] Sun Hyeok Lee,[a,c] Naren Raja,[c,g] Hui-suk Yun,[c,g] Jeong-Soo Lee*[b,c,f] and Chang-Soo Lee*[a,c]

[a]Hazards Monitoring BNT Research Center, Korea Research Institute of Bioscience and Biotechnology (KRIBB), Daejeon 34141, Republic of Korea
[b]Disease Target Structure Research Center, Korea Research Institute of Bioscience and Biotechnology (KRIBB), Daejeon, 34141, Republic of Korea
[c]Department of Biotechnology, University of Science & Technology (UST), Daejeon 34113, Republic of Korea
[d]Department of Chemical Engineering and Applied Chemistry, Chungnam National University, Daejeon 34134, Republic of Korea
[e]Department of Polymer Engineering, Chonnam National University, Gwangju 61186, Republic of Korea
[f]Dementia DTC R&D Convergence Program, Korea Institute of Science and Technology (KIST), Seoul 02792, Republic of Korea
[g]Powder and Ceramics Division, Korea Institute of Materials Science (KIMS), Changwon 51508, Republic of Korea

§ The authors contributed equally to this work.
* Corresponding author: (J.-S.L.) jslee@kribb.re.kr; (C.-S.L.) cslee@kribb.re.kr

Table S1 Reaction conditions and optical characterization for carbon dots (CDs).

	Reaction reagents			UV lamp images in DW (at 365 nm)		Excitation (nm)	Emission (nm)	Product
	Alendronate sodium trihydrate	Ethylenediamine (EDA)	DI-water	Vis/UV				
**a**	1.0014 g	0 μL	40 mL	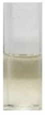	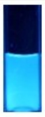	330	410	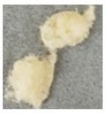
**b**	1.0026 g	335 μL	40 mL	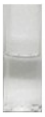	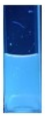	340	405	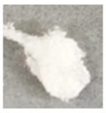
**c**	1.0020 g	500 μL	40 mL	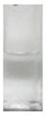	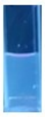	330	400	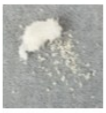
**d**	1.0031 g	1000 μL	40 mL	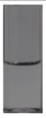	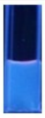	320	400	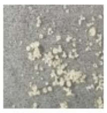
**e**	1.0017 g	2000 μL	40 mL	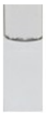	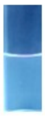	295	395	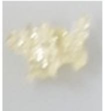

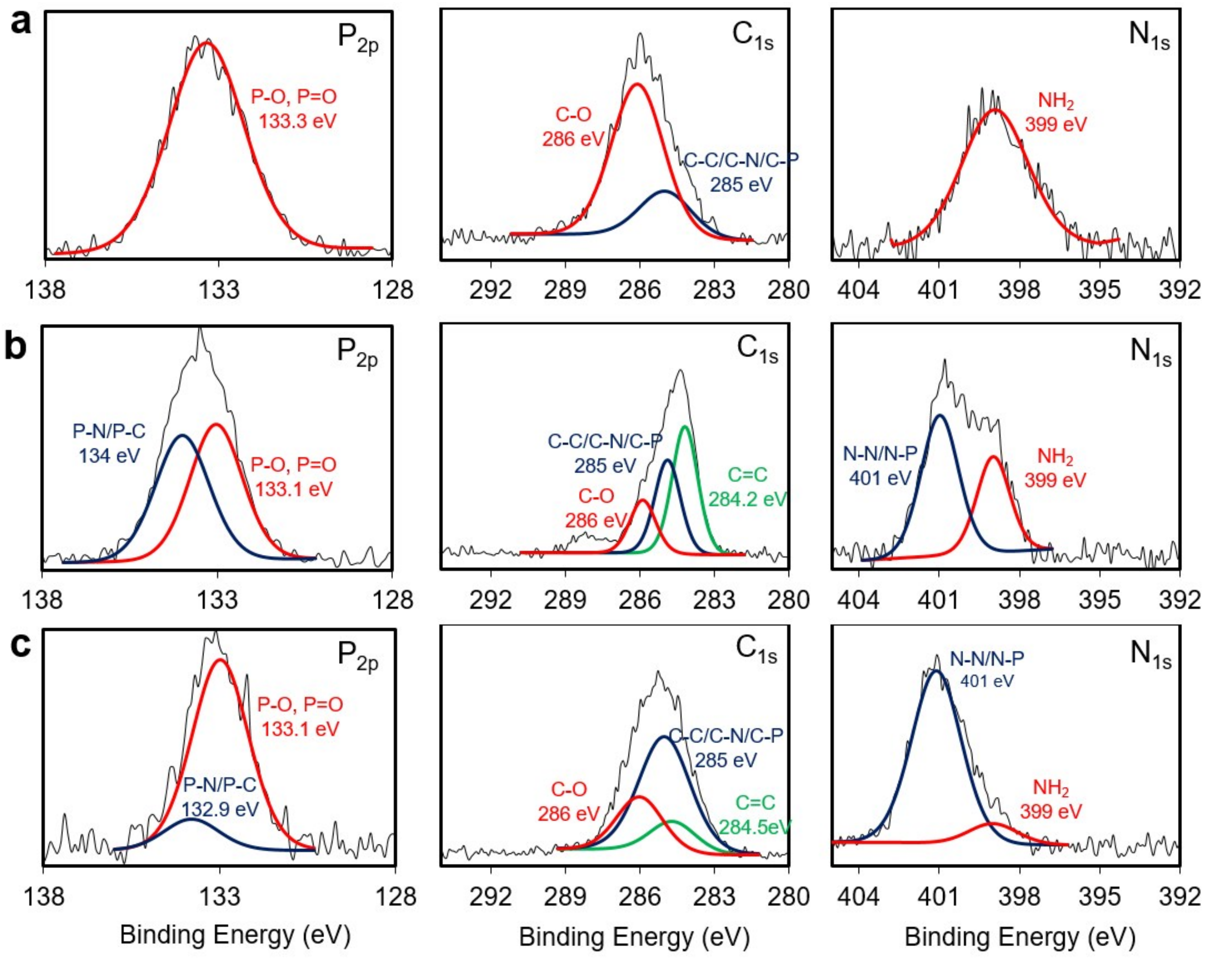

Fig. S1 X-ray photon spectroscopy (XPS) analysis of a) Alendronate, b) Alen-EDA-CDs, and c) Alen-CDs.

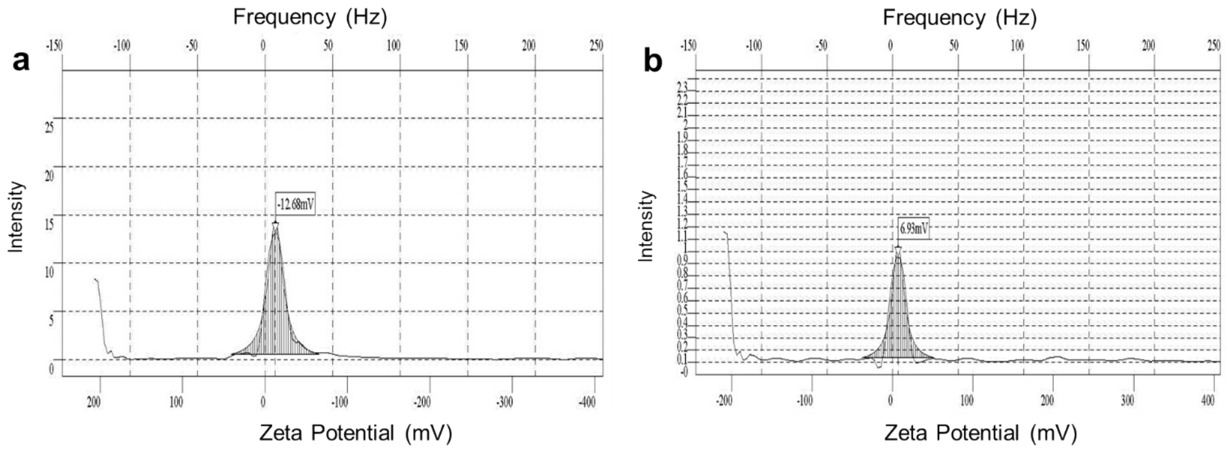

Fig. S2 Distribution of mobility analysis of a) Alen-CDs and b) Alen-EDA-CDs using zeta potential (n=5).

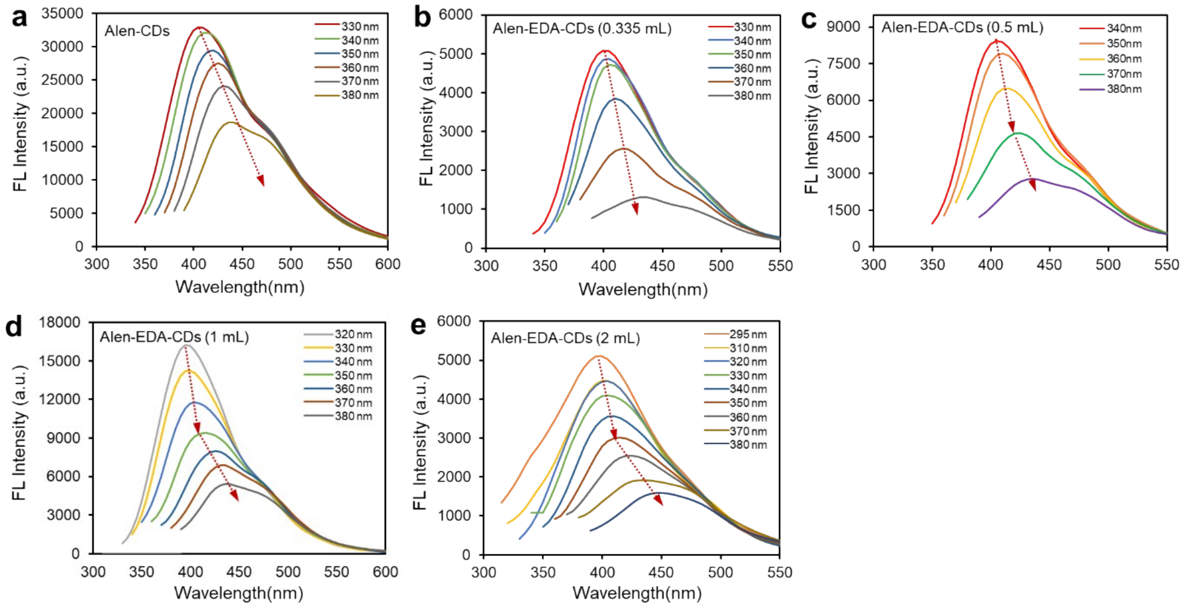

Fig. S3 Excitation dependence of fluorescence spectrum of a) Alen-CDs and Alen-EDA-CDs doped with EDA at b) 0.335 mL, c) 0.5 mL, d) 1 mL and, e) 2 mL.

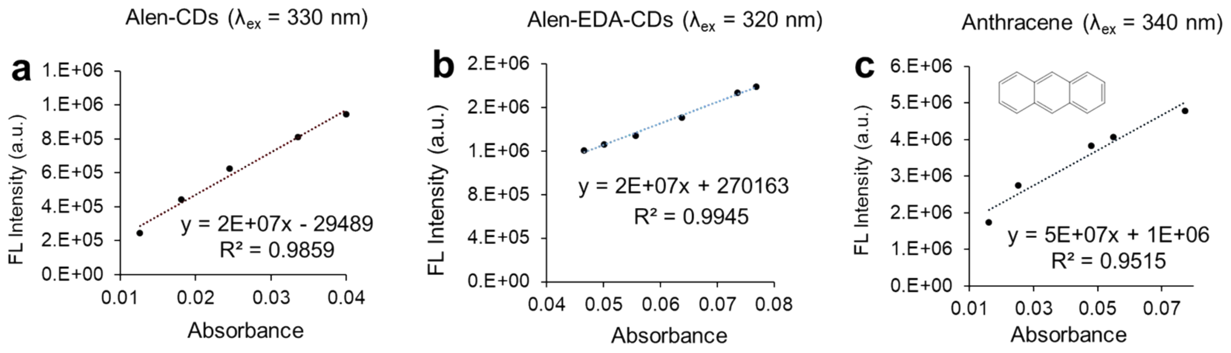

	A-CD in DI water (RI = 1.3330)
Slope	2E+07x (24,971,878)
QY	13.5 %

	Alen-EDA-CDs in DI water (RI = 1.3330)
Slope	2E+07x (19,690,666)
QY	10.6 %

	Anthracene in ethanol (RI = 1.3614)
Slope	5E+07x (47,887,140)
QY	27 %

Fig. S4 Quantum yield of a) Alen-CDs, b) Alen-EDA-CDs, and c) anthracene as a relative reference fluorophore.

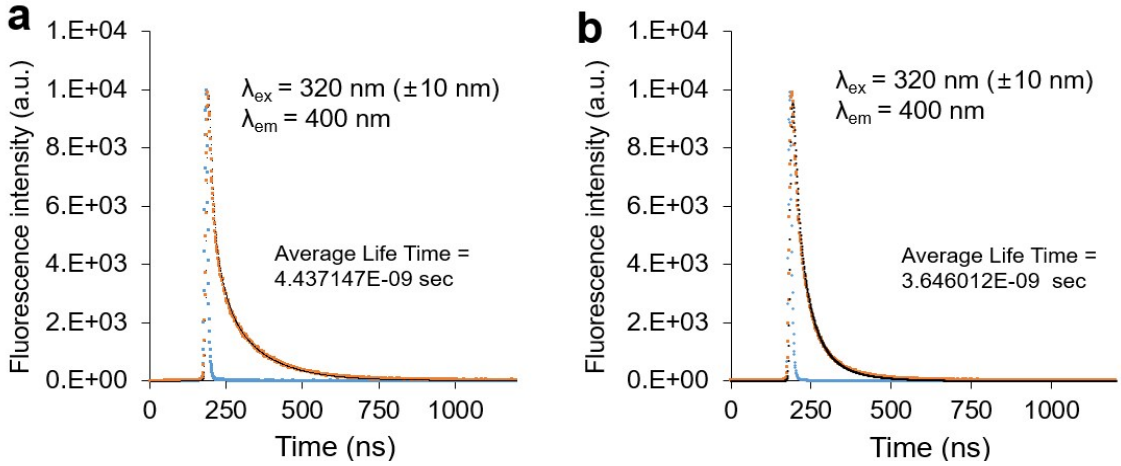

Fig. S5 Fluorescence decay profiles of a) Alen-CDs and b) Alen-EDA-CDs at 320 nm excitation and 400 nm emission.

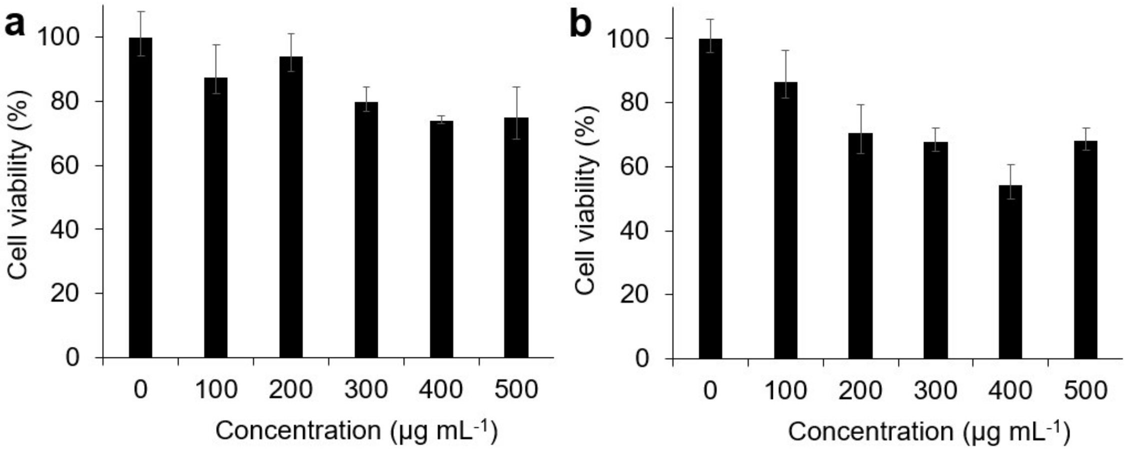

Fig. S6 Cells viability on HeLa cell of a) Alen-CDs and b) Alen-EDA-CDs (n=5).

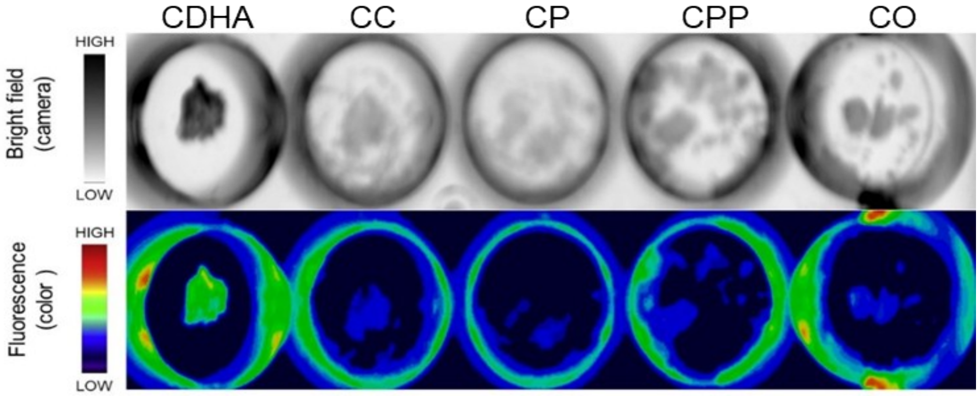

Fig. S7 Specific affinity of Alen-CDs for calcium deficient hydroxyapatite (CDHA), calcium carbonate (CC), calcium phosphate (CP), calcium pyrophosphate (CPP), and calcium oxalate (CO).

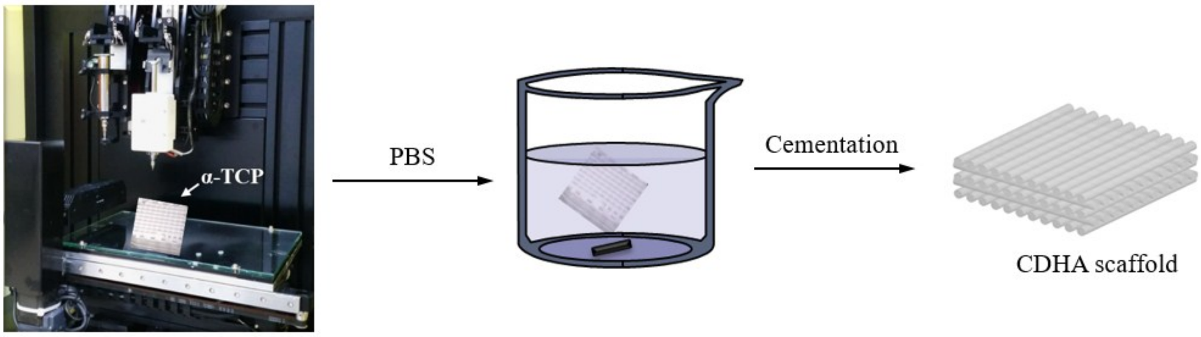

Fig. S8 Schematic diagrams of CDHA scaffold fabrication.

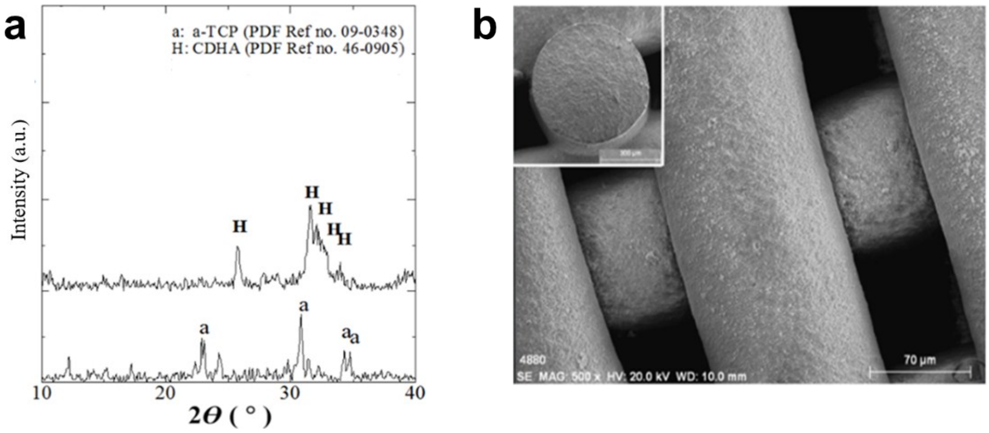

Fig. S9 a) XRD result of α-TCP scaffold (before cementation) and CDHA scaffold (after cementation), and b) SEM image of CDHA scaffold (insert is closed section).

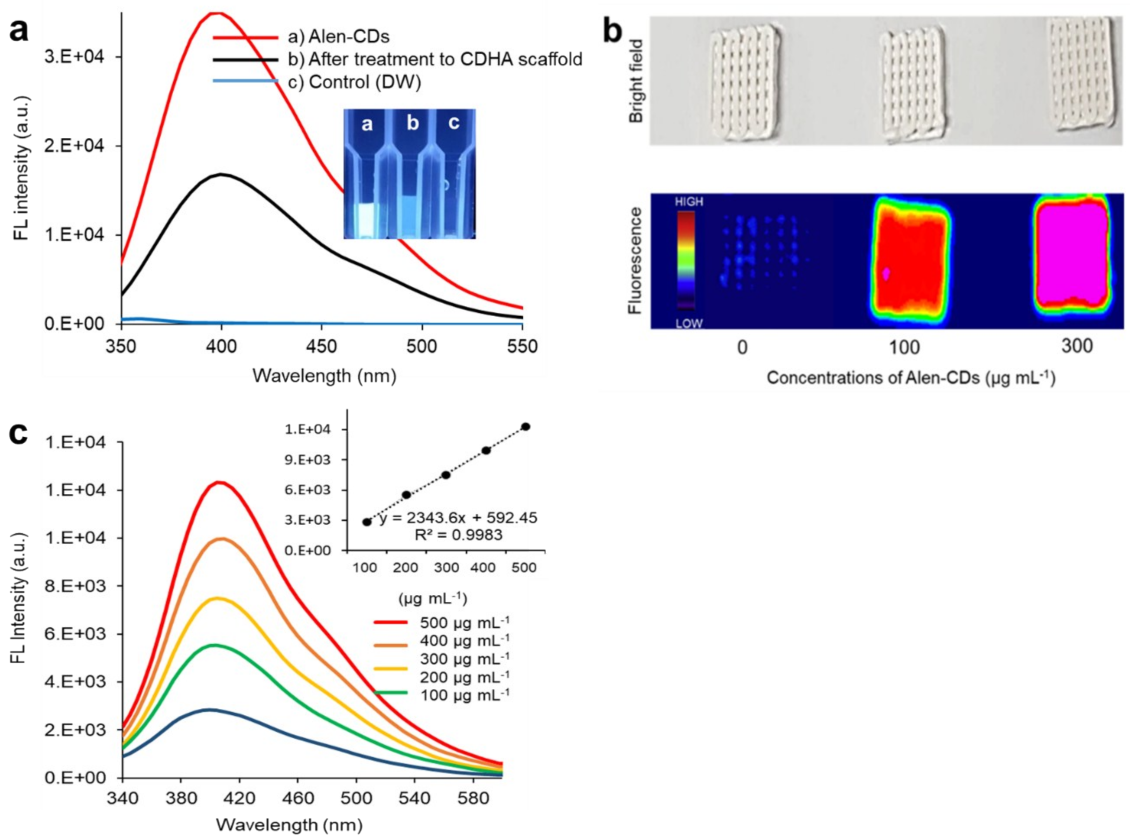

Fig. S10 a) Fluorescence spectrum and images of Alen-CDs solutions (before and after adsorption), b) fluorescence images of Alen-CDs treated scaffolds at different concentrations (0, 100, 300 μg mL^{-1}), and c) calibration curve for emission spectrum of Alen-CDs treated scaffolds at different concentrations (100–500 μg mL^{-1}).

Table. S2 EDAX analysis of CDHA scaffold a) before and b) after Alen-CDs treatment.

a Before Alen-CDs treatment

Element	Weight %	Atomic %
O	15.57	29.75
P	26.11	25.77
Ca	58.31	44.48
Totals	100.00	

b After Alen-CDs treatment

Element	Weight %	Atomic %
C	11.26	18.50
N	2.18	3.07
O	45.19	55.73
P	16.13	10.27
Ca	25.25	12.43
Totals	100.00	
